# Microbial gene expression analysis of healthy and cancerous esophagus uncovers bacterial biomarkers of clinical outcomes

**DOI:** 10.1038/s43705-023-00338-1

**Published:** 2023-12-05

**Authors:** Daniel E. Schäffer, Wenrui Li, Abdurrahman Elbasir, Dario C. Altieri, Qi Long, Noam Auslander

**Affiliations:** 1 Computational Biology Department, Carnegie Mellon University, Pittsburgh, PA 15213 USA; 2 The Wistar Institute, Philadelphia, PA 19104 USA; 3 University of Pennsylvania, Philadelphia, PA USA; 4 Department of Cancer Biology, University of Pennsylvania, Philadelphia, PA 19104 USA; 5 Present Address: Massachusetts Institute of Technology, Cambridge, MA 02139 USA

**Keywords:** Microbiology, Gastrointestinal diseases

## Abstract

Local microbiome shifts are implicated in the development and progression of gastrointestinal cancers, and in particular, esophageal carcinoma (ESCA), which is among the most aggressive malignancies. Short-read RNA sequencing (RNAseq) is currently the leading technology to study gene expression changes in cancer. However, using RNAseq to study microbial gene expression is challenging. Here, we establish a new tool to efficiently detect viral and bacterial expression in human tissues through RNAseq. This approach employs a neural network to predict reads of likely microbial origin, which are targeted for assembly into longer contigs, improving identification of microbial species and genes. This approach is applied to perform a systematic comparison of bacterial expression in ESCA and healthy esophagi. We uncover bacterial genera that are over or underabundant in ESCA vs healthy esophagi both before and after correction for possible covariates, including patient metadata. However, we find that bacterial taxonomies are not significantly associated with clinical outcomes. Strikingly, in contrast, dozens of microbial proteins were significantly associated with poor patient outcomes and in particular, proteins that perform mitochondrial functions and iron-sulfur coordination. We further demonstrate associations between these microbial proteins and dysregulated host pathways in ESCA patients. Overall, these results suggest possible influences of bacteria on the development of ESCA and uncover new prognostic biomarkers based on microbial genes. In addition, this study provides a framework for the analysis of other human malignancies whose development may be driven by pathogens.

## Background

Esophageal carcinoma (ESCA) is among the most common cancers, with around 600,000 new cases diagnosed each year [1, 2]. The five-year survival rate for esophageal cancer patients is low, with estimates ranging across populations from 15% to 24%, and is markedly lower than the survival rates of patients with other common gastrointestinal cancers, such as stomach (21–33%) and colon (59–71%) cancers [3]. While some lifestyle factors, such as smoking, are known to contribute to the development of ESCA, the causes and risk factors remain incompletely characterized [2]. Like other organs of the gastrointestinal tract, the healthy esophagus has a substantial resident bacterial population, principally members of *Streptococcus* and a handful of other genera [4, 5]. Yet, shifts in the esophageal microbiome have been associated with the development of esophageal cancer and of a precursor condition called Barrett’s esophagus [6]. Beyond microbiome shifts, several bacterial species in the colon are thought to be oncogenic in colorectal cancer, such as *Streptococcus bovis*, *Bacteroides fragilis*, and *Fusobacterium nucleatum* [7, 8]. *F. nucleatum* is also a pathogenic member of the oral microbiome, where it may promote development of oral squamous cell carcinomas [8, 9]. It is therefore possible that bacteria in the esophagus are oncogenic or protective, and such bacteria will likely demonstrate cancer or healthy tissue specific presence patterns.

The most accessible data for studying the tumor microenvironment are short-read transcriptome (RNAseq) data. In addition to studying the presence of organisms, these data can provide insight into the complement of microbial proteins that are expressed in an environment [10]. However, RNAseq reads are typically very short, introducing several challenges to analysis of diverse bacterial species [11]. For example, RNAseq reads in The Cancer Genome Atlas (TCGA) are typically 48 or 75 nucleotides. The length and abundance of microbial reads make *de novo* assembly of longer coding sequences extremely challenging [11, 12]. Methods for read identification without assembly, using alignment [13] or other sequence search approaches, rely on databases of sequenced organisms. However, the size of microbial databases poses a computational challenge for such approaches, which are limited in precision by the short length of each sequence [11, 12].

Despite these limitations, screening large volumes of cancer RNAseq reads, such as those included in TCGA, for sequences of likely microbial origin has been used to identify varied and complex bacterial populations of tumors [14–16]. Comparisons between samples taken from tumors and nearby non-cancerous tissue have shed further light on the differences between tumor and adjacent microenvironments, revealing diverse microbial species with shifted prevalence in cancer [17, 18]. In a comparative study of several cancer types, ESCA had a high abundance of bacterial reads, consistent with other GI tract cancers, but among the lowest prevalence of fungal reads [17]. These studies have focused on data from only cancer patients in TCGA or similar datasets; however, tumor-adjacent tissues are not necessarily healthy [19], and may not capture the full range of variation between healthy and cancer microbiota.

Here, we extend to bacterial sequences our approach for fast assembly of microbial RNAseq reads into longer contigs [20] and apply it to provide, for the first time, systematic comparison of bacterial populations in esophageal cancer and in healthy esophagus. We obtain RNAseq reads from ESCA samples in TCGA [21] and esophagi of healthy individuals in the Genotype-Tissue Expression (GTEx) dataset [22]. We train a new convolutional neural network to discriminate bacterial, viral, and human sequences to predict reads of likely bacterial origin, reducing the burden for assembly. We then assemble putative microbial sequences guided by those predictions, providing longer sequences for more accurate identification of microbial species and genes. We identify dozens of bacterial genera that are significantly over- or underrepresented in cancer. In addition to identifying bacterial taxa, we find several bacterial proteins whose expression is associated with poor patients’ survival, and study host gene expression patterns associated with these proteins. These analyses give further insight into the striking differences in the esophageal microbiome of healthy individuals and cancer patients, and allow estimation of specific pathways and mechanisms through which the altered expression of bacterial proteins may be associated with oncogenesis.

## Methods

### Model training

To classify reads, we trained a model to predict the origin of a 76-base pair sequence from among human, viral, and bacterial. To simulate RNAseq reads from each class, we segmented into 76-base sequences (1) the human hg19 reference transcriptome, obtained from NCBI [23], 2) a database of transcripts from diverse viruses of placental mammals, obtained from the Virus Variation Resource [24], and (3) a database of bacterial genomes containing one representative per genus, curated previously [25]. To generate balanced data, sequences were segmented with stride two for viral sequences, stride 26 for human sequences, and stride 130 for bacterial sequences. Sequences were randomly divided into training, validation, and testing sets; this split was done before segmenting. Segments containing N’s were excluded. This yielded a training set of size 21,005,972 (7,000,098 human, 6,996,574 viral, 7,009,300 bacterial), a validation set of size 4,503,578 (1500036, 1498065, 1505477), and a testing set of size 5,628,298 (1873416, 1863322, 1891560). To predict the likely origin of reads, we trained a small convolutional neural network, with two convolutional layers and one fully-connected layer (Supplementary Text). We tuned most hyperparameters and selected the best-performing model by one-versus all area under the precision-recall curve (AUPRC) on the validation set. All models were trained using TensorFlow 2.8 [26].

### Sequence assembly and identification

We obtained 75-base RNAseq reads from 170 esophageal carcinomas through TCGA [21] and 76-base reads from 1565 healthy esophageal samples from 742 unique individuals through GTEx [22]. These projects used similar RNAseq protocols [27, 28]; briefly, total RNA was isolated, polyadenylated RNAs were enriched (eukaryotic mRNAs are 3′ polyadenylated), cDNA was synthesized from the RNA, amplified, and purified, and reads were sequenced using the Illumina HiSeq 2000. We first removed reads that map to the human genome using the hg19 reference. We then obtained model scores assigned to each read, denoting the relative likelihoods of human, viral or bacterial origins. For prediction and assembly, we excluded all reads with more than one N (0.17% of unmapped TCGA reads; 0.57% of unmapped GTEx reads). Overall, we considered 2,656,993,271 TCGA reads and 631,388,801 GTEx reads. For reads with one N (0.22% of unmapped TCGA reads; 3.74% of unmapped GTEx reads), we replaced the N with a random nucleotide for prediction only. We also padded TCGA reads, again for prediction only, with a random 3′ nucleotide to match the 76-base length expected by the model. On the validation data, we found that replacing only one or two nucleotides with a random replacement had only a small impact on model performance (Supplementary Fig. S1).

Once human, bacterial, and viral model scores were assigned to each read, we used those predictions to guide assembly of the reads into larger sequences. We considered every read with a bacterial or viral score of at least 0.46 to be a “seed” read (Supplementary Fig. S2). To prioritize sequences that were (1) likely to be microbial and (2) likely to be bacterial, we sorted the seed reads to first take likely bacterial seeds in descending bacterial score order and then likely-viral seeds in descending viral score order. For each seed, we attempted to assemble a longer sequence by greedily extending the seed in each direction using a modification of the assembly tool developed previously [20] (Supplementary Text). For assembly, we considered an N to match any nucleotide and, when such a match happened during extension, kept the non-N nucleotide.

### Mapping assembled microbial sequences to bacterial taxa

We identified the resulting putative microbial species present in each sample by comparing them to several curated databases of microbial nucleotide sequences using blastn [29]. For bacterial sequences, we used the set of NCBI representative bacterial genomes (approximately one per bacterial species). We additionally used two databases of viral RNA sequences, one for ‘reference’ human viruses and the other for ‘novel’ or non-human viruses, curated in our previous work [20]. We filtered hits with e-value below 0.01 and assigned the sequence and species from the top BLAST hit to each sequence. For characterizing the abundance of organisms in cancer, we pooled all species at the genus level to reduce the number of hypotheses and to reflect the possible inaccuracy of identifying short sequences at the species level.

### Over and under representation of microbial genera

We then compared the prevalence of bacterial genera in ESCA and healthy esophagus. We computed the prevalence of each genus in each sample, pooling all species in each genus. We also pooled occurrences in multiple esophagus samples from the same patient. Overall, we identified at least one bacterial transcript in all 161 ESCA cases and in healthy esophagus samples from 742 distinct patients. We selected as genera of interest those that occurred in at least 10% of ESCA or 10% of healthy samples. To quantify bacterial over- or underabundance in cancer, we performed a one-tailed binomial test, using the binom_test method from scipy 1.10 [30]. For each genus, we set the hypothesized probability to be the fraction of healthy samples in which the genus was detected, except that we used minimum and maximum probabilities of 0.0001 and 0.9999, as using exactly 0 or 1 would always produce a *p*-value of 0. We then specified the number of successes as the number of ESCA samples in which the genus was detected, the number of trials as 161, and the hypothesis as “less” or “greater” depending on whether the ESCA abundance was lower or higher than the healthy abundance. We corrected the *p*-values using Benjamini–Hochberg FDR correction [31].

### Confounder corrected analysis for over and under representation of microbial genera and proteins

In addition to the analysis described above, we performed a similar analysis when correcting for possible confounders, such as clinical and background differences between TCGA and GTEx cohorts. We therefore used 715 individuals from GTEx and 122 cases from TCGA with complete background information to perform the analysis (that is, with race, age, sex, weight, and smoking information). We additionally included the sequencing depth of each sample as a cofounder in the corrected analysis, using the average sequencing depth for individuals with multiple samples. We employed chi-squared test, which is appropriate for this large dataset with hundreds of samples. To adjust for confounders, we first fitted a boosted logistic regression model with confounders as covariates to estimate the probabilities of being in the TCGA vs GTEx cohorts. The resulting AUC (area under the curve) was 1.00, indicating substantial differences between the cohorts based on these confounders. Then, we performed weighted Chi-squared tests to evaluate bacterial under and over representation, where the weights are the inverse of estimated probabilities of being in the TCGA vs GTEx groups. In the weighted data, the covariates are balanced between the TCGA and GTEx groups. Therefore, using the weighted chi-squared test allows us to mitigate confounders in the evaluation of bacterial under and over representation in TCGA vs GTEx groups. For this analysis, we considered all bacterial genera with any abundance. We then used FDR correction [31] to correct for multiple hypotheses.

We used an identical approach to perform a corrected analysis for the over- or underprevalence of microbial protein families, which were identified as described below.

### Phylogenetic analysis

We created a tree of selected bacterial genera by obtaining 16S rRNA gene sequences, one per genus, from GenBank, choosing a RefSeq sequence if available. We then aligned these sequences using MUSCLE version 5.1 [32, 33] with default parameters, and constructed a tree using FastTree version 2.1.11 [34] with default parameters. The tree was visualized using iTOL [35].

### Survival analyses

To evaluate the association between bacterial species and ESCA survival we correlated the presence of each individual species (for which at least 5 positive and 5 negative ESCA samples were identified; excluding samples with no clinical data) with overall and disease stable survival using the log-rank test through Python lifeline package [36]. TCGA clinical information was obtained through the TCGA Clinical Data Resource [37]. This (meta)dataset includes, among other measures, both overall survival, which measures time to the death of a patient, and disease-free survival, which measures the time until cancer recurs after primary therapy. Log-rank *p*-values estimating association between expression of different bacterial genera and overall and disease-free survival were FDR-corrected for multiple comparisons, where no significant association was found (Supplementary Text). To evaluate the association between microbial proteins and survival, we similarly compared overall and disease-free survival for patients positive and negative for the expression of each microbial protein (for which at least 5 positive and 5 negative ESCA samples are identified). We identified several microbial proteins that were significantly associated with survival after FDR correction for multiple comparisons (Supplementary Text).

### Mapping assembled contigs to microbial genes

We mapped the assembled contigs to microbial genes through RefSeq non-redundant microbial sequence database, downloaded from NCBI through the non-redundant proteins annotated on representative genomes. Contigs were mapped using blastx, with e-value below 1e-5. Presence or absence of each microbial gene in each sample considered were used for further analysis. For these analyses, we considered 155 of the 170 ESCA samples with available clinical information. Where healthy esophagus contigs were used, we considered all 1565 samples.

### Host gene expression analyses

To evaluate host correlates of microbial iron-related (Fe) genes, we analyzed human gene expression data of TCGA ESCA samples. RNAseq RSEM values for ESCA samples were downloaded from cBioportal [38, 39]. We compared the expression of all human genes between samples positive vs those negative for microbial Fe proteins that were found significantly associated with poor outcomes (accessions WP_006680945.1, WP_002532908.1 and WP_131625607.1) using a rank-sum test. None of the genes were significantly associated with microbial Fe-gene presence after FDR correction for multiple comparisons. To evaluate the processes that were upregulated in these samples, we extracted human genes assigned with unadjusted *p*-value < 0.05, and where the median z-score for Fe-positive samples was above 0.2, and that for Fe-negative samples was below 0. We used KEGG enrichment [40] to identify host (human) pathways enriched with genes upregulated in microbial Fe-positive ESCA samples.

### Genome scale metabolic modeling

To compare oxygen consumption and ATP production rates between ESCA samples that are positive or negative for microbial genes associated with poor survival, we used genome scale metabolic modeling (GSMM). We used the GIMME algorithm [41] to constrain each metabolic model by the gene expression values in each ESCA sample, and applied Flux Balance Analysis (FBA) [42] to generate a predicted metabolic flux for each sample. We used the Recon1 human metabolic model [43] and the COBRA Toolbox v.3.0 implementation of GSMM functions [44].

## Results

**Fig. 1:**
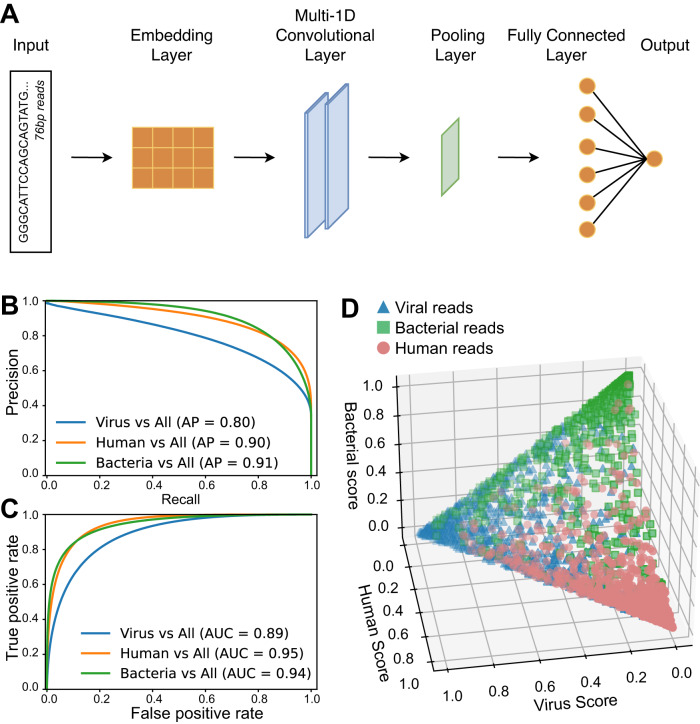
Read-classification model architecture and performance. **A** Overview of the model architecture. **B** Test-set one-versus-all precision-recall curves for each class of sequence origin. **C** Test-set one-versus-all receiver-operating characteristic curves for each class. The AUCs are the areas under each curve. **D** Model scores for 1000 randomly-selected sequences from each class, plotted on the *x* + *y* + *z* = *1* plane.

To allow alignment free prediction of viruses and bacteria from short-read RNAseq data, we first trained a convolutional neural network to classify 76-base nucleotide sequence as having human, viral, or bacterial origins (Fig. 1A). To simulate RNAseq reads for training, we used segmented sequences from the human transcriptome, viral transcriptomes, and bacterial genomes (“Methods”). We trained dozens of convolutional neural networks with varying hyperparameters and selected the model with the best performance on a held-out validation set. We then evaluated our final model on a separate test set of held-out human, viral, and bacterial sequences (Fig. 1B–D). It demonstrated one-versus-all Area Under the Precision-Recall Curve (AUPRC) of 0.89 for human sequences, 0.91 for bacterial sequences, and 0.80 for viral sequences. The best possible AUPRC is 1.0, corresponding to a perfect classifier, while the AUPRC of a random classifier is equal to the fraction of positive examples, which is about 0.33 in the balanced three-class case. The model further demonstrated Area Under the Receiver-Operating Curve (AUROC) of 0.95 for human sequences, 0.94 for bacterial sequences, and 0.89 for viral sequences. The best possible AUROC is 1.0, corresponding to a perfect classifier, while the AUROC of a random classifier is 0.5.

The model serves as the first step of the pipeline to identify bacterial and viral pathogens from RNAseq data. Starting with unmapped RNAseq reads, predictions from the model are used to guide assembly into longer putative-pathogenic contigs. Then, these contigs are aligned to broad databases of viral and bacterial genomes to detect those that are expressed in each sample. We applied this pipeline to study the prevalence of viruses and bacteria in esophageal cancer, using RNAseq data from cancer patients (obtained via TCGA) as well as from a larger population of healthy control esophagi (obtained via GTEx). Using the labeled contigs produced by the pipeline, we first searched for bacterial genera that are under or overrepresented in cancer.

**Fig. 2:**
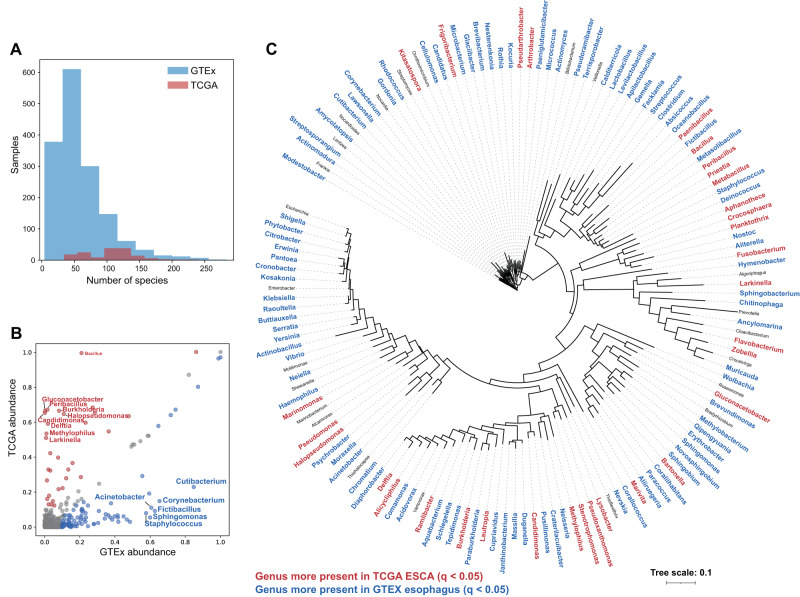
Bacterial genera over- and underabundant in esophageal carcinoma vs healthy tissues. **A** A histogram of the numbers of district bacterial species detected in each ESCA (TCGA, red) and healthy (GTEx, blue) sample. **B** A scatterplot of the abundance in ESCA and healthy esophagus of each bacterial genera; genera with sufficient representation and with significant differences are colored red if overabundant in ESCA and blue if underabundant in ESCA. Genera with 50 percentage-point differences in abundance are labeled. **C** A 16S rRNA-based tree of bacterial genera with sufficient representation in ESCA or healthy esophagus. Genera that are significantly overabundant in ESCA are shown in red, and genera that are significantly underabundant in ESCA are shown in blue.

Overall, we attributed sequences from 161 ESCA cases and 742 healthy esophagi to 6,961 unique bacterial species (Fig. 2A, Supplementary Data S1, S2). Considering 145 genera that are sufficiently represented in the data (Methods, Fig. 2B), and applying a permissive threshold for presence of one contig, we found 32 genera that were significantly overprevalent in cancer and 90 that were significantly under-prevalent in cancer (*p*_FDR_ < 0.05; Fig. 2B, C, Supplementary Fig. S3, Supplementary Data S3). We additionally performed this analysis controlling for possible confounders and differences between the cohorts, including the sequencing depth of each sample (“Methods”; Supplementary Data S4). The cancer underabundant bacterial genera are particularly notable, as the read depth and number of species found were both lower for the GTEx samples compared to TCGA samples, despite lower sequencing depth (Fig. 2B). Because of the sample size, even small absolute differences in abundances can be significant (Fig. 2B).

We note the genera with the largest absolute differences as best distinguishing the cancer and healthy conditions. Among the 90 underabundant genera, four occur in at least 50 percentage points fewer ESCA samples than healthy: *Cutibacterium*, *Sphigomonas*, *Fictibacillus*, and *Corynebacterium* (Fig. 2B, C). The family *Sphingomonadaceae*, which includes *Sphigomonas*, was previously suggested to be protective against breast cancer [45]. The highlighted bacterium in that study was a member of the genus *Sphingobium*, which we find in 18.3% of healthy esophagi but only a single ESCA sample (Fig. 2B, C). Additionally, *Corynebacterium parvum* was first reported to promote an immune response and survival in cancer more than 40 years ago [46, 47].

Among the 32 overabundant genera, nine occur in at least 50 percentage points *more* ESCA samples than healthy: *Bacillus*, *Gluconacetobacter*, *Peribacillus*, *Candidimonas, Burkholderia*, *Delfita*, *Halopseodomonas*, *Methylophilus*, and *Larkinella* (Fig. 2B, C). Most of these genera occur in a very small fraction of healthy esophagi and a bit more than half of ESCA samples. However, most striking is the common genus *Bacillus*, which was detected in all but one ESCA sample for which any bacterial sequences were detected, but only 21% of healthy esophagi. Aside from the closely-related *Bacillus* and *Peribacillus*, as well as the unique *Larkinella*, the other genera six genera represent *Alpha*-, *Beta*-, or *Gamma*-*Proteobacteria*. Interestingly, increased *Proteobacteria* abundance was previously reported in pancreatic and breast cancers [48, 49], and was previously reported in nine cancer types from TCGA [50]. At the genus and clade level, these increases of common taxa may represent an overall increase in bacterial load in ESCA, or may be linked to tissue and microenvironment differences between the cohorts. On the other hand, members of the small genus *Larkinella* (class *Cytophagales*), which have been isolated from diverse environments, principally soil [51–55], were identified by one study in bladder cancer, reporting an association between *Larkinella* and recurrence [56].

Interestingly, we found very low levels of *Helicobacter* (including *H. pylori*) in both GTEx samples (0.1%) and TCGA samples (0.6%). This supports the specificity of *H. pylori* as an oncogenic agent in stomach cancer only, and is consistent with previous studies and meta-analyses finding either no or a weak negative (protective) association between overall *H. pylori* infection and ESCA [57, 58].

In addition to bacteria, we also examined the presence of viral clades in with ESCA and healthy tissues. Overall, we found matches to 691 unique viral strains in 61 ESCA samples and 503 healthy esophagi (Supplementary Data S5–S7). The most common clade observed is herpesviruses, which were detected in 32 ESCA samples and 162 healthy esophagi. Strikingly, we observed a *Geobacillus* bacteriophage in 192 healthy esophagi, where 181 were positive for type E2 and 98 were positive for type E3. Interestingly, however, *Geobacillus* bacteriophage was not detected a single ESCA sample. Surprisingly, we directly detected *Geobacillus* in only 17 esophagi, and detected both *Geobacillus* and a *Geobacillus* phage in only four esophagi. This could be explained by a possible different host of this bacteriophage, or enhanced expression of the bacteriophage compared to the bacterial host. Of additional note is a virus of the genus *Vientovirus*, DNA viruses that infect *Entamoeba gingivalis* [59] and are found in the human mouth and respiratory tract [60], found in two ESCA samples.

Previous studies have suggested that the presence of specific bacteria in several tumors is correlated with survival [61–63]. Given the number of cancer overabundant genera, we hypothesized that the same might be true for ESCA. We therefore searched for bacterial species whose presence or absence in tumor RNAseq is correlated with the survival of ESCA patients (see “Methods”). However, no significant associations were found.

We reasoned that instead of the presence of a specific bacterial taxon, microbial processes executed by different bacteria may be associated with oncogenesis and therefore correlated with outcomes. This would be consistent with the large number of overabundant bacterial clades yet lack of species correlated with patient survival. We therefore turned to identifying specific microbial proteins that are expressed in ESCA and evaluating whether any such proteins correlate with outcomes.

**Fig. 3:**
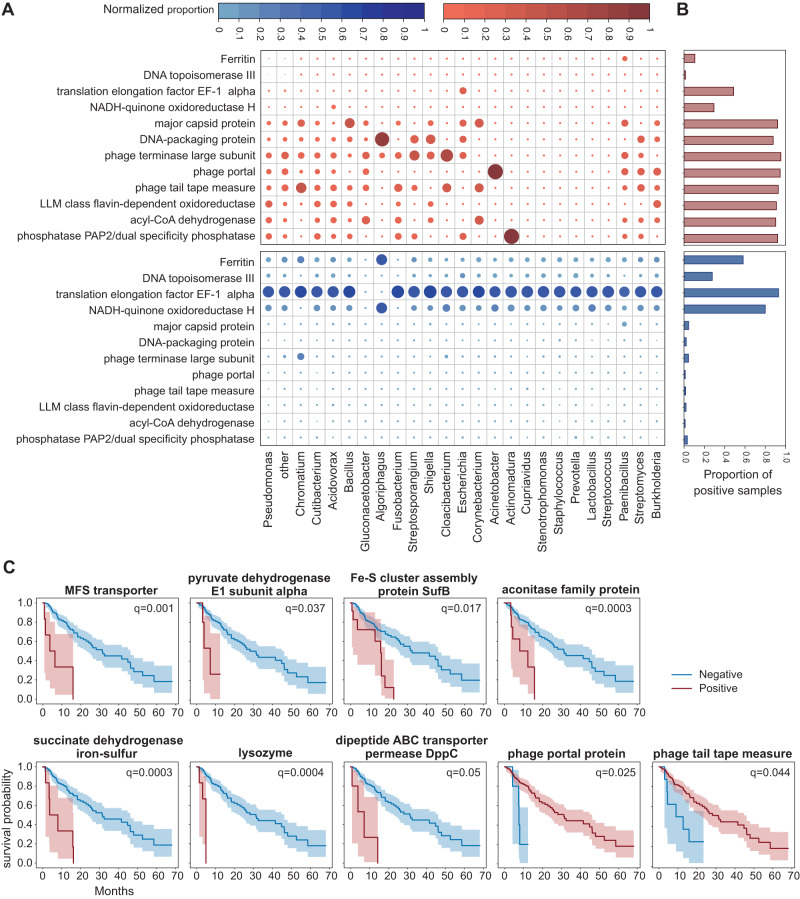
Microbial genes associated with progression free survival. **A** Circle heatmaps showing the normalized proportion of samples positive for microbial genes (y-axis) from different bacteria (x-axis) in ESCA cancer (upper panel, in red) and normal esophagus (bottom panel, in blue). Proportions are normalized so the values in each column sum to 1, i.e., each (protein, genus) value indicates the proportion of samples positive for any of the proteins from that genus that are positive for the given protein. **B** Bar plots showing the overall proportion of each bacterial gene, from all species, in ESCA cancer (red) and normal esophagus (blue) samples. **C** Kaplan Meier curves comparing the DSS between ESCA patients positive (red) and negative (blue) for each bacterial gene. The log-rank *p*-value is reported for significant associations with FDR-corrected *q* < 0.05.

To that end, we mapped each microbial contig against a database of representative microbial proteins. Among all samples, we identified transcripts of 16,261 bacterial proteins, including transcription products of several notable gene families from diverse bacteria in both healthy and cancerous samples (Fig. 3A, B, Supplementary Data S8). As expected, the large majority (87.6%, *N* = 14248) had little difference in prevalence between cancer and healthy (at most a 5-percentage-point difference in ESCA and healthy occurrences). However, some protein families did show considerable differences in prevalence. Only 21 were substantially more present in healthy esophagus (healthy frequency – ESCA frequency > 25%). The top five include translation elongation factor EF-1 alpha, ferritin, NADH-quinone oxidoreductase subunit H, and two unnamed protein products comprising nucleotide-binding domains. The healthy-abundant proteins also include a zincin-like metallopeptidase protein and DNA topoisomerase III, which are present in only 1.3% and 0.6% of ESCA samples, respectively, and several transposases. In contrast, 697 proteins were comparably overrepresented in the cancer samples (ESCA frequency – healthy frequency > 25%). This asymmetry may be explained in part by the greater sequencing depth of ESCA samples – the average protein is present in 2.7% more ESCA samples than healthy esophagi. Most strikingly, phage replicative proteins are consistently more abundant in cancers (Fig. 3A, B), and the top overpresent proteins in ESCA (occurring in 80 percentage points more ESCA samples, *N* = 66) include at least 37 phage protein families. While many of these hits may be redundant, at least 7 phage components are represented in the top proteins. Other top cancer-abundant proteins include an acyl-CoA dehydrogenase, an LLM-class flavin dependent oxidoreductase, ABC transporter components, multiple peptidases including the S49 family, and multiple phosphatases (Fig. 3A, B, Supplementary Data S8). We additionally found that, overall, more than 2000 protein families are significantly (*q* < 0.05) differentially present after controlling for possible confounders and differences between the cohorts, including the sequencing depth of each sample (Methods; Supplementary Data S9).

Among the bacterial gene families found expressed in cancer samples, several are significantly associated with overall and disease stable survival of patients (Fig. 3C, Supplementary Data S10). In particular, there are 34 families whose presence in the sample is significantly negatively associated with survival, although several were phage, ribosomal, or unlabeled proteins (Supplementary Data S10). Among the remainder, MFS transporters, of which we found hits to three representatives among the 34 families, comprise a diverse and ubiquitous class of multi-substrate membrane transport proteins [64, 65]. While MFS transporters have a clinically-important role in antibiotic resistance [65, 66], their possible role in human cancers has not been elucidated. Specifically, removal of chemotherapy agents in drug-resistant cancers is generally performed by ABC transporters rather than human MFS homologs [66]. Lysozyme is a small antibacterial protein that principally targets bacterial cell walls, especially those of Gram-positive bacteria [67, 68]. While it is primarily known as a multifunctional component of animal immunity [67], lysozyme is produced by many organisms, including bacteria [68], for microbial defense and competition.

Among the microbial proteins that are significantly associated with survival, several are linked with mitochondrial functions, such as pyruvate dehydrogenase, succinate dehydrogenase and aconitase. This implies a possible metabolic shift in cancers expressing these microbial proteins, linked with enhanced complex II respiration and oxidative stress. Indeed, examining host gene expression, oxidative phosphorylation gene expression is elevated in samples positive for these microbial proteins (Supplementary Fig. S4A). Furthermore, using genome scale metabolic modeling (“Methods”) we find that oxygen consumption rates and ATP production are elevated in ESCA samples expressing these microbial proteins, supporting the notion that mitochondrial shift may be underlying the link between these proteins and poor patients’ outcomes (Supplementary Fig. S4B, C).

Three protein families that are significantly associated with poor survival are microbial iron-sulfur cluster proteins: aconitase, succinate dehydrogenase iron-sulfur, and iron-sulfur cluster assembly SufB. Indeed, iron is required for bacterial proliferation [69, 70]. Therefore, we investigated whether the presence of these genes was correlated with changes in the human tumor transcriptome.

**Fig. 4:**
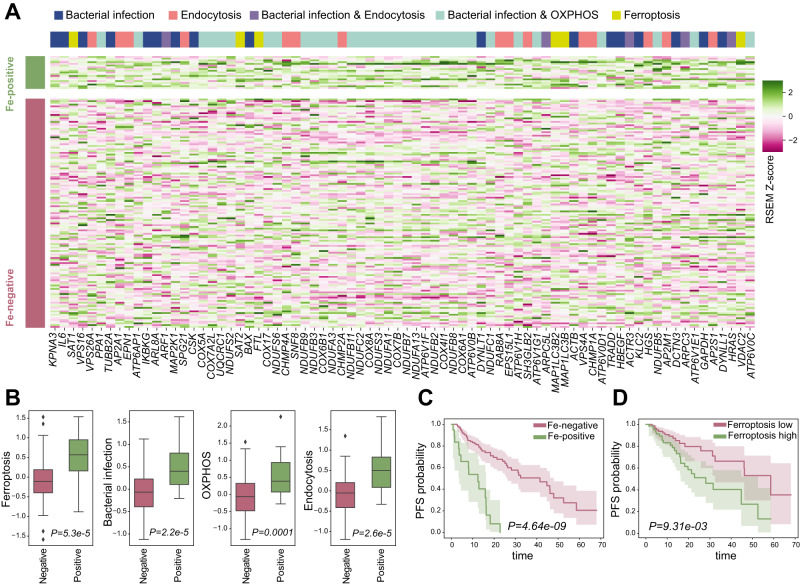
Host upregulated pathways in ESCA samples positive for Fe-genes. **A** heatmap showing the gene expression (RSEM Z-score) of human genes upregulated in Fe-genes positive samples, belonging to four pathways significantly upregulated. **B** Boxplots comparing the average gene expression of genes in the four pathways between Fe-genes positive and negative samples. **C** Kaplan Meier curves comparing the PFS between ESCA patients positive vs negative to any of the Fe-genes, and right panel **D** the PFS between ESCA patients with high vs low average ferroptosis gene expression level (using the median as threshold).

We identified a large number of upregulated host genes in ESCA samples expressing microbial iron proteins, across four key upregulated pathways: bacterial infection response, endocytosis, oxidative phosphorylation, and ferroptosis (Fig. 4A, B; Supplementary Data S11). Ferroptosis, in particular, is a recently-characterized cell death pathway, with relevance to cancer progression [71]. Previous research has also identified differential expression of ferroptosis-pathway genes in ESCA, although the exact set of genes identified differs [72]. As observed with the individual gene families, presence of bacterial Fe-genes overall is negatively associated with survival (Figs. 3C and 4C). Further, high expression of distinct host ferroptosis genes is itself associated with worse survival, in contrast to the three other pathways (Fig. 4D, Methods). These genes include *SAT1*, *SAT2*, *FTL*, *MAP11C3B2*, *MAP1lC3B*, and *VDAC2*. Increased *SAT1* expression, including by the p53 tumor suppressor, promotes the ferroptosis cell death pathway [73]. *SAT1* and *SAT2* regulate polyamine metabolism, a process which has long been implicated in cancer [73, 74]. Indeed, higher expression of the *FTL* ferroptosis regulator, is associated with a poorer prognosis in hepatocellular carcinoma [75]. Further, expression of the voltage-gated channel *VDAC2* is also associated with increased risk in some cancers. *VDAC2* is also a target of erastin, a small-molecule promotor of ferroptosis in cancer cells [76, 77]. However, interestingly, expression of *SAT1* as well as *SAT2* has been linked to improved outcomes in several adenocarcinomas [78–81]. We therefore evaluated the association of SAT1 and SAT2 with survival individually, but found that lower expressions of SAT1 and SAT2 individually do not correlate with survival (Supplementary Text).

## Discussion

Several lines of emerging evidence point to a substantial role of tumor and resident microbes in cancer development and progression [82–84]. Bulk tumor RNA sequencing can be utilized to study both intratumor and tumor-microenvironment microbial expression. However, existing short-read RNA sequencing datasets, which represent the largest source of cancer sequence information, are ill-suited for researching microbiomes. In particular, short nucleotide reads are very challenging to map accurately to individual microbial species or specific proteins. The naïve alternative to direct read mapping is an exhaustive assembly of sequencing reads to produce longer putative contigs, but this is computationally infeasible for all but the smallest sequencing datasets. Further, knowledge of a cancer microbiome has very limited diagnostic or prognostic value without comparison to a suitable non-cancerous control. While paired comparisons between cancer and nearby non-cancerous tissue are the most straightforward, microbiome disruptions that precede cancer may occur in nearby non-cancerous tissue as well. For example, canonical oncogenic viruses generally lead to cancer only after a persistent, often decades-long infection of the tissue of origin [85–87], which is likely to be widespread relative to the cancer cell of origin.

Here, we developed a new method based on the rationale of our previous approach for virus identification, viRNAtrap [20], to overcome many of these challenges in the characterization of bacterial populations from RNAseq. We then applied it to compare bacterial species and proteins in esophageal carcinoma (ESCA) and the healthy esophagus. To overcome the limitations of both direct mapping and naïve assembly, our approach first employs a deep learning model to identify RNAseq reads with likely bacterial or viral origin. We then used those as seeds in a targeted seed-and-extend assembly pipeline to produce longer candidate microbial contigs. These contigs were then mapped to curated databases of bacterial and viral nucleotide sequences, as well as bacterial protein families. To understand patterns in the ESCA microbiome at the population level, we used comparable RNAseq samples from hundreds of healthy esophagi as a robust non-cancerous control.

We found substantial differences in the complements of bacterial taxa and bacterial protein products between ESCA samples and the healthy population. Most genera with nontrivial prevalence in one population were present at significantly different rates, with the majority more abundant in healthy esophagi. Yet, surprisingly, we did not identify genera whose presence is significantly correlated with outcome among the ESCA patients. In contrast, most bacterial protein families with a significant difference in prevalence were more commonly detected in cancers, although this might be attributable to variations in sequencing depth enabling the detection of proteins with a lower level of expression in the ESCA samples.

Surprisingly, about half of the top bacterial proteins that we identify as overexpressed in cancer are derived from phages. While the role of the bacteriophages encoding these proteins is unclear, bacteriophages may alter microbiomes by disproportionally infecting certain bacterial species and by facilitating gene transfer [88]. It is therefore plausible that certain combinations of phages could favor cancer-associated bacteria. We identified several bacterial protein families whose presence is also associated with outcomes in ESCA patients. We further found that bacterial expression of iron-sulfur proteins in ESCA was associated with altered expression of host genes. The affected human genes included several in the ferroptosis pathway, an alternate cell death pathway, that was independently associated with poor outcomes. One possible mechanism to link ferroptosis dysregulation with poor patient outcomes is through iron excess and ferroptosis resistance, supported by upregulation of FTL, which stores iron and is upregulated in ferroptosis resistant cells [89]. Excess iron beyond iron storage capacity allows for redox-active iron and oxidative stress [90]. Indeed, several microbial genes associated with ESCA outcomes confer mitochondrial functions and were linked with host oxidative phosphorylation. Importantly, mitochondrial oxidative phosphorylation is increasingly recognized as a key mechanism for metabolic reprogramming in cancer [91, 92]. Collectively, these findings suggest that methods to study cancer microbiomes that produce only a species identification, such as 16S rRNA sequencing, are insufficient for completely understanding potential microbial contribution to cancer and for development of microbial biomarkers.

While we observe multiple significant association both with the cancer state and clinical outcomes, it is important to note that causal role in oncogenesis may not be inferred through such correlative analysis. Local microenvironment conditions and other clinical or behavioral factors can modulate both microbiome and esophageal cancer, and therefore underlie the observed differences. Comparison between unrelated cancer and healthy populations eliminates pre-cancer infections in the control samples. However, possible differences between the populations and experimental contaminants may affect the observed patterns in such comparison. The successes of the prediction and assembly steps are somewhat variable, and also depend in part on the read lengths. While in many cases we can extend model-selected reads to form longer contigs, the assembly does not always produce a longer contig, and correspondingly better species and protein identifications. Especially, this approach is less likely to capture lowly expressed microbial elements. The direct tradeoff of this approach is that, in exchange for selecting reads and obtaining longer contigs, we are unable to obtain a reliable measure of expression levels for the microbial species or proteins identified. While we can still use the underlying RNAseq dataset to perform quantitative analyses of the host transcriptome, we are now constrained to binary (presence vs absence) analyses of the microbial transcriptome. Yet, our approach can be easily adjusted to rapidly estimate total bacterial and viral load from RNAseq.

As with any sequencing data, there is a possibility of microbial contamination during the sequencing process. To reduce this risk, we screen out a list of known, common contaminants (Supplementary Text). There is also a possibility that the microbial reads recovered by the RNA sequencing performed for GTEx and TCGA are not representative of the overall sample microbiome, both because of general sequencing biases and because the sequencing pipelines used were optimized for eukaryotic mRNAs rather than bacterial or viral RNAs. Additionally, while our extended approach covers both bacteria and viruses, it currently does not handle other components of the cancer microbiome. Principally, these are likely to include fungal and other eukaryotic pathogens, some of which have been implicated in cancers [17]. Despite these limitations, we are still able to identify with both high throughput and high precision microbial genes in existing RNAseq datasets.

### Supplementary Information

Supplementary Text

Supplementary table S1

Supplementary table S2

Supplementary table S3

Supplementary table S4

Supplementary table S5

Supplementary table S6

Supplementary table S7

Supplementary table S8

Supplementary table S9

Supplementary table S10

Supplementary table S11

## Data Availability

The results shown here are in whole or part based upon data generated by the TCGA Research Network: https://www.cancer.gov/tcga. The raw FASTQ RNA sequencing data are protected and are not publicly available due to data privacy laws but are available under restricted access as data can be unique to an individual. Access can be obtained from the Genome Data Commons (GDC) after receiving permission via dbGaP, following the steps described in https://www.ncbi.nlm.nih.gov/projects/gap/cgi-bin/study.cgi?study_id=phs000178.v11.p8. The processed data including microbes identified and respective statistics are available as Supplementary Information. The complete data generated in this study are provided in the Supplementary Information/Source Data file.

## Code availability

All code and scripts associated with this work are publicly and freely available through GitHub: https://github.com/AuslanderLab/virnatrap-bacteria.
