# Supplementary material for: Microbial gene expression analysis of healthy and cancerous esophagus uncovers bacterial biomarkers of clinical outcomes
Source: ISME Commun. 2023 Dec 5;3:128. doi: 10.1038/s43705-023-00338-1 (PMC10696091; doi:10.1038/s43705-023-00338-1)
Supplement: Supplementary file 1 — Supplementary Text [file 43705_2023_338_MOESM1_ESM.docx]

**Supplementary Text**

*Model training: detailed model architecture and training procedures*

We trained a convolutional neural network, consisting of an embedding layer, two 1D convolutional layers with 64 filters each of width 64 and padding with zeros, a max-pooling layer with width 9 (and stride 1), one fully connected layer with 64 units, all with ReLU activation, and an output layer with SoftMax activation. The learning rate was set to 0.0001, and L2 normalization with weight 0.01 was used.

During training, we performed hyper-parameter tuning over the number of convolutional layers and units, the number of fully connected layers and units, the width of the convolutions, and the width of the max pool. We also performed limited tuning of the learning rate and dropout. Models were compared based on validation-set one-versus-all area under the precision-recall curve (AUPRC).

All models were trained using TensorFlow 2.8 for 100 epochs using the Adam optimizer, treating the number of epochs as a hyperparameter. Most hyperparameter tuning was performed by training models on a randomly-selected quarter of the training dataset, which we observed to produce only a marginal decrease in training-set performance. Additionally, during hyperparameter tuning, approximately 4,000 sequences containing ambiguous nucleotides other than N, all encoded as A, were erroneously included in the training data. The final model was retrained on the full training set and with sequences containing ambiguous nucleotides excluded.

*Sequence assembly and identification: handling N’s in sequences*

*Sequence assembly and identification: assembling sequences from seed reads*

For each seed read, we attempted to assemble a longer sequence by greedily extending the seed in each direction using a modification of the assembly tool developed for viRNAtrap. Specifically, we searched for the terminal 24-mer of the current sequence in all other reads, and then, if at least one match was found, extended with the matching read that gave the largest extension.

All matching reads were considered consumed and ineligible for inclusion into another sequence. Additionally, any reads that were found to be wholly contained in each contig were excluded from any future contig. Where applicable, an N was considered to match against any nucleotide, and when an N was aligned against another nucleotide in the assembly on a contig the non-N was always kept.

*Survival analyses: association of bacterial species and proteins with survival*

All survival analyses were performed by comparing the presence vs. absence of each bacteria species or protein. Significance was evaluated using the log-rank test, through Python lifelines.statistics.StatisticalResult v0.27.4. P-values were FDR-corrected for multiple comparisons. Survival curves were fitted and visualized using Kaplan Meier curves, through Pythom lifelines.fitters.kaplan_meier_fitter.KaplanMeierFitter.

*Non-associations of host genes with patient survival*

The ferroptosis host genes that are upregulated in bacterial Fe-positive samples include *SAT1* as well as *SAT2* which have been linked to improved outcomes in several adenocarcinomas. We therefore applied a similar survival analysis, using the expression of *SAT1, SAT2* and the z-score combining *SAT1* and *SAT2*, all of which were not significantly associated with survival. We conclude that *SAT1* and *SAT2* are not individually associated with better survival in ESCA, and that their combined expression with the other ferroptosis host genes identified is associated with poor survival.

*Identifying common sequencing contaminants*

We used the list of collected contaminants, including vector contaminants and different sequence artifacts that were identified previously for viRNAtrap. These were used to filter out assembled contigs from being mapped to microbial species or genes. Any accessions associated with contaminants were entirely removed from the search.

**Supplementary Figures**

**
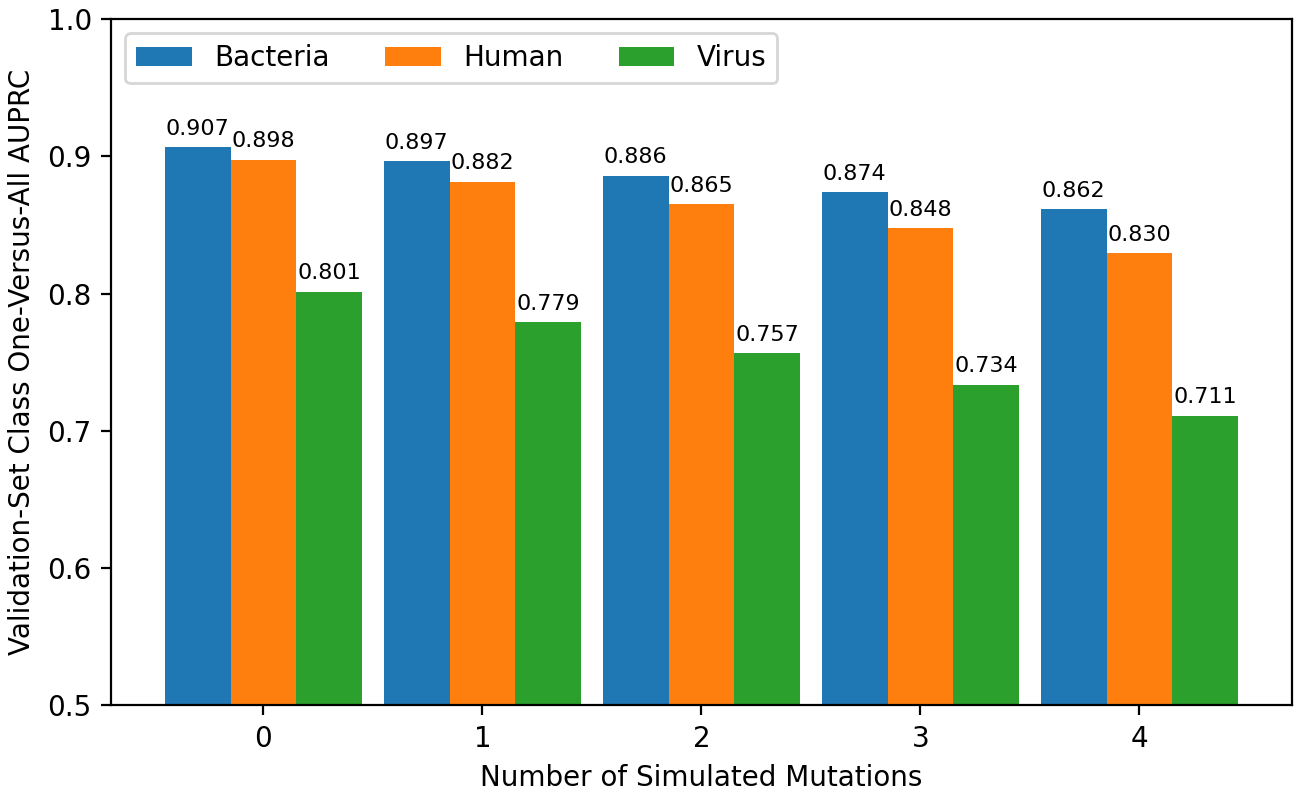
**

**Figure S1.** **Effect of random mutation on model performance.** To understand the effect of including reads containing N’s, as well as reads that were padded from 75bp to 76bp, on our pipeline, we examined the performance of the classification model on reads from the validation set with 0, 1, or 2 randomly-selected bases changed to a different nucleotide. Class one-versus-all AUPRCs are shown for 0-4 random mutations for each of bacterial, viral, and human simulated reads. We found that, with one mutation, class one-versus-all AUPRCs were reduced by 0.016 for human, 0.010 for bacteria, and 0.022 for virus. With two mutations, AUPRCs were reduced by 0.032, 0.021, and 0.045, respectively. We assessed this to be a relatively small impact in performance, especially as we expect to correctly replace an N 25% of the time on actual reads, and therefore included RNAseq reads with at most one N in our pipeline as well as using our 76-basepair model on 75-bp TCGA reads rather than retraining a 75-bp model. Further mutations had a roughly linear increasing impact on performance, as shown.


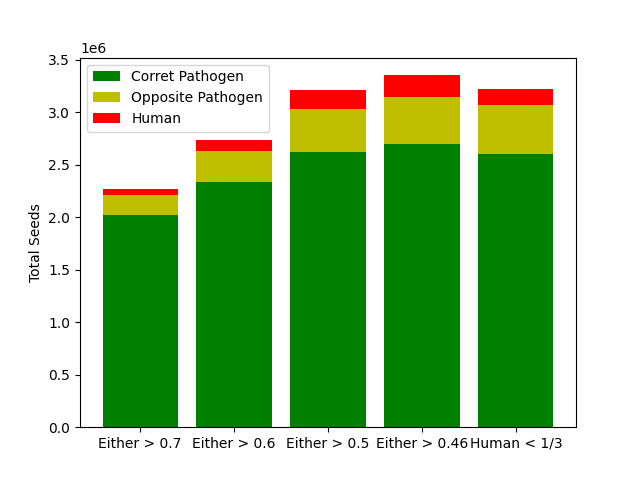


**Figure S2. Comparison of “seed” read score thresholds.** The number of test-set simulated sequences that would be selected as a “seed,” in millions, based on the model scores and one of five possible thresholds. The first four thresholds describe a minimum value on either the bacterial or viral scores. The last threshold describes a maximum threshold on the human score. Reads that pass each threshold are categorized as correct pathogen (bacterial/viral reads whose bacterial/viral score is highest; green), opposite pathogen (bacterial/viral reads whose viral/bacterial score is highest; yellow), and human reads (red).

*
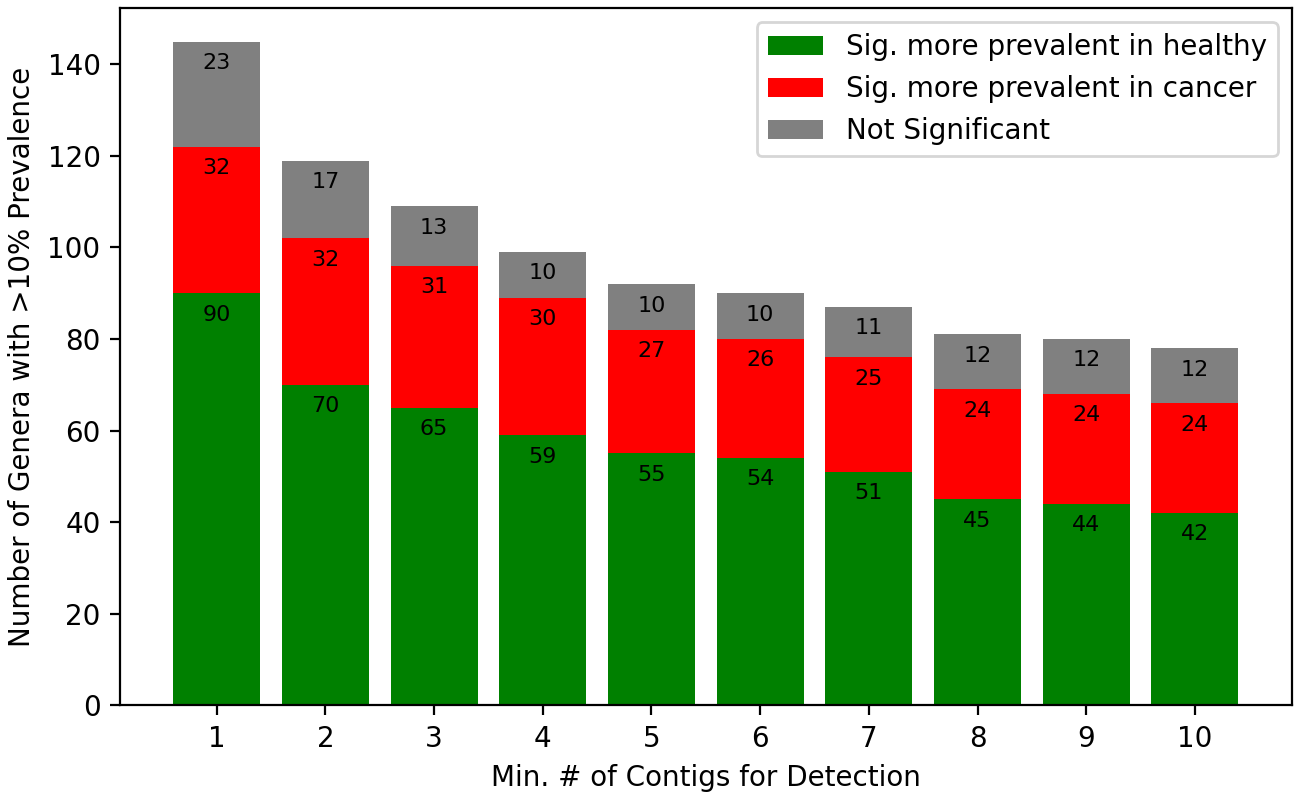
*

**Figure S3. Number of genera detected with varying contig thresholds.** The number of bacterial genera that are found in at least 10% of GTEx or TCGA esophageal samples, where “found” is defined as assigning a minimum of k reads to a sequence from that genus, for values of k between 1 and 10. Genera are grouped by whether they are significantly over-prevalent in GTEx samples (green, binomial *p*_FDR_ < 0.05), over-prevalent in TCGA samples (red), or not significant in either direction.

**Figure S4. Host metabolic shift associated with microbial protein presence in ESCA samples.** **(A)** Oxidative phosphorylation genes that are upregulated in ESCA samples positive for microbial proteins. **(B)** and **(C)** violin plots comparing the predicted flux (using genome scale metabolic modeling) in ATP generating reactions **(B)** and oxygen consuming reactions **(C)**. The rank-sum p-values are reported.

**Supplementary Data**

**Data S1. Assignments of bacterial proteins and species to putative microbial contigs from TCGA ESCA samples.** Columns are: 1) a unique contig ID, 2) a bacterial protein accession, 3-12) The following parameters from blastx: pindent, qcovs, length, mismatch, gapopen, qstart, qend, sstart, send, and evalue, 13-14) invariant TCGA project identifiers, for all ESCA samples 15) TCGA patient identifier, 16) TCGA tissue description, 17) assigned bacterial name, and 18) a bacterial genome accession. For completeness, TCGA samples include adjacent healthy, although these samples were excluded from the analyses.

**Data S2. Assignments of bacterial proteins and species to putative microbial contigs from GTEx esophagus samples.** Columns and format are the same as in **Data S1**, except columns 13-16 all contain the GTEx sample identifier. Samples from the same patient can be identified by the second token (i.e., GTEX-XXXXX) of the sample identifier.

**Data S3. Presence of bacterial genera in ESCA or GTEx esophagus.** Columns are: 1) Genus, 2) proportion of TCGA samples with the, 3) proportion of healthy esophagi with the genus, 4) *p*-value of a significant difference in presence in ESCA (see Methods), 5) FDR-corrected *p*-value, 6) Indicator of whether TCGA prevalence is greater than healthy prevalence, 7) Indicator of whether defined as sufficiently prevalent (at least 10% in healthy or ESCA), 8) FDR-corrected *p*-value considering only 10%-prevalent genera, 9-11) prevalence in healthy samples from each esophageal region.

**Data S4. Results of metadata-corrected prevalence analysis on all bacterial genera.** Columns are: 1) Genus, 2) Uncorrected *p*-value; 3) FDR-corrected *p*-value.

**Data S5. All viral species matches to putative microbial contigs from ESCA samples.** Columns are 1-2) TCGA case accession, 3) virus accession 4) TCGA project ID, 5) TCGA sample type.

**Data S6. All viral species matches to putative microbial contigs from GTEx samples.** Columns and format are the same as in Data S5 columns 1 and 3.

**Data S7 Abundance of viruses in ESCA or GTEx esophagus.** Columns are as in Data S3, except that there is no consideration of a viral abundance threshold, so columns 6-7 in S3 have no ortholog.

**Data S8 All bacterial proteins identified in at least one ESCA or GTEx sample, with abundances in each.** Columns are: 1) the protein family accession, 2) the frequency in ESCA samples, 3) the frequency in healthy esophagi, 4) a “score” comparing the frequencies, 5) the log base 2 of the score, 6) the absolute difference between the ESCA and healthy frequencies. The score is computed based on the ratio between frequencies as (1+ESCA freq)/(1+healthy freq). The log2 of the score varies between -1 and 1, with 0 corresponding to no difference and higher values corresponding to higher frequencies in ESCA. 7) Protein names.

**Data S9. Results of metadata-corrected prevalence analysis on all bacterial proteins.** Columns are: 1) the protein family accession, 2) Uncorrected *p*-value; 3) FDR-corrected *p*-value.

**Data S10. Association p-value of each bacterial protein found in ESCA with patient survival**. Ratios and FDR *q*-values for each protein for both overall survival (OS) and disease-specific survival (DSS).

**Data S11. List of host (human) genes upregulated in the presence of bacterial Fe-S proteins.** Columns are: 1) Gene names, 2) Median z-score in Fe-negative samples, 3) Median z-score in Fe-positive samples. For all genes, the median z-score for Fe-positive samples was above 0.2, and that for Fe-negative samples was below 0.
